# What Makes a Product Sustainable? A US‐Based Consumer Survey Finds That Consumers Overemphasize the Impact of Packaging on Product Sustainability

**DOI:** 10.1111/1750-3841.71511

**Published:** 2026-10-10

**Authors:** Jonas Ahonen, Anna Wagner, Hannah Mulheron, Alissa Nolden, Emily J. Mayhew

**Affiliations:** ^1^ Department of Food Science and Human Nutrition Michigan State University East Lansing Michigan USA; ^2^ Department of Food Science University of Massachusetts Amherst Amherst Massachusetts USA

## Abstract

Understanding the drivers behind consumers’ purchasing decisions is crucial to supporting the sustainability of the global food system. This study examined consumers’ sustainability‐related beliefs, attitudes, and behaviors regarding food and its packaging among a US‐based cohort (*n* = 475). Participants responded to an online survey including: (1) a maximum difference test for food product attributes; (2) sustainability tension scenarios between two food products; (3) a section asking participants how often they make certain sustainable purchasing decisions; (4) a section asking participants to rate their level of agreement with sustainable statements; and (5) a demographic questionnaire. We identified significant gaps in consumer perceptions of sustainability. Findings revealed consumers consistently prioritize highly visible attributes like recyclability and packaging material, while assigning lower value to the environmental impact of the food product itself. Furthermore, results indicated considerable resistance to adopting plant‐based alternatives compared to meat and dairy products, alongside difficulty in identifying the sustainability benefits of shelf‐life extension and food waste reduction methods. These results suggest that consumers have a narrow view of sustainability, focused primarily on recycling and end‐of‐life packaging, which causes consumers to overlook other, potentially more impactful, aspects of sustainability. Instead, targeted education that emphasizes the benefits of alternative protein sources and the functional role of preservatives in reducing food waste is essential to growing a truly sustainable food system.

## 1 Introduction

The food system is a major factor in global environmental change, contributing significantly to greenhouse gas emissions (GHG), deforestation, biodiversity loss, and pollution across stages of production, distribution, consumption, and disposal. The agri‐food system is responsible for 29.7% of global GHG emissions, which have increased by 10% since 2000 (FAO 2024). Food waste is a major contributor to this problem; in 2020, food waste in landfills was responsible for 55 million metric tons of CO_2_ equivalents and approximately 58% of methane emissions from landfills (Krause et al. 2023). In addition to these emissions, 39% of Earth's ice‐free land is used for agriculture (Ellis et al. 2010), contributing to deforestation and loss of natural carbon capture. Reducing GHG emissions from the food system is essential to achieving global sustainability goals and supporting climate change initiatives.

While innovations in food production systems must be part of the solution, changing consumer behavior is another essential component for mitigating environmental impacts of the food system. The importance of consumer behavior and education in meeting sustainability goals is widely recognized. The 17 Sustainable Development Goals were agreed upon by United Nations member states in 2015 as a universal call to protect the planet's environment and the people of it by 2030 (UNDP 2016). Among these goals is an emphasis on the importance of Quality Education in sustainable development.

Sustainability has rapidly grown into an issue of public relevance, with sustainability education becoming more widespread to increase consumer literacy on the environmental impacts of their choices (Bauer and Morlock 2026). With increasing pressure from consumer activists, some companies are updating their practices and communications to reflect sustainability values, although not all changes drive meaningful improvements in environmental impact—a trend characterized by the term “green‐washing” (Brown et al. 2026). Gong et al. (2023) found that sustainability messaging in marketing led to increased consumer engagement and improved brand image, showcasing further benefit for companies to adopt sustainable practices.

When it comes to consumers’ role in reducing the negative environmental impacts of food production and consumption, consumers often face ambiguity in terms of which product choices have the greatest impacts on the environment. For instance, Dlamini et al. (2024) found that consumers often overestimate the sustainability of glass packaging, viewing it as more sustainable than plastic alternatives. In actuality, glass can have a higher carbon footprint due to its increased weight and energy required in transportation (Dlamini et al. 2024). Similarly, consumers perceive packaging waste as having a higher carbon impact than food waste, despite food waste having a substantial impact on GHGs (Brennan et al. 2023). While public service campaigns and national consumer education initiatives have emphasized recycling to increase sustainability, far less outreach is focused on reducing food waste (Barnett et al. 2023).

Previous research on consumer perceptions of the sustainability of food packaging has highlighted the complexity of consumers' behaviors, attitudes, and understanding of sustainability. Systematic literature reviews indicate that consumers express interest in making sustainable choices, yet they lack the information needed to understand how their food choices impact sustainability (Van Bussel et al. 2022). Importantly, sustainability remains a secondary priority, with food purchasing primarily driven by price, taste, and convenience rather than sustainability characteristics (Van Bussel et al. 2022).

A 2023 review (Cook et al. 2023) concluded that increasing sustainability labeling on food products could be an effective tool in promoting sustainable choices, but recent studies have shown that consumers have a poor understanding of what greenhouse gas emissions labels mean (Meyerding et al. 2019) and that labels with unfamiliar or technical sustainability information may result in information overload (Rondoni and Grasso 2021). The effectiveness of sustainability labels may therefore depend not only on the availability of information, but also on consumers’ ability to understand and interpret the information presented. Arrazat et al. (2023) found that when carbon footprint labeling was articulated with a color‐coded traffic light symbol, consumer choices shifted toward products with lower carbon footprints. This simplified labeling allows for quick and intuitive comparison of sustainability between products. Furthermore, color‐coded labels increased purchase intention for products with positive environmental impact and decreased purchase intention for products with negative environmental impact (Taillie et al. 2024). These findings suggest that providing sustainability information in a simpler manner may be effective in promoting sustainable purchasing decisions.

To push society toward more sustainable purchasing and consumption habits, we must first understand consumer perceptions about which food purchasing behaviors are more or less sustainable and how these values drive purchasing decisions. Although previous research has examined consumer perceptions of sustainability labels, packaging, and food, less is known about how consumers prioritize sustainability across multiple food product and packaging attributes, particularly when these attributes involve competing sustainability factors. Targeted consumer education can bridge these gaps in consumers’ perceptions of sustainability and actual environmental impact. Identifying where these gaps occur is important for developing targeted consumer education and communication strategies that address sustainability factors beyond the highly visible characteristic of food packaging. Only when these gaps are identified, targeted consumer education can begin to close them.

The specific objectives of this study were:

1. Identify which food product attributes consumers consider important or unimportant for sustainability.
2. Characterize how consumers evaluate trade‐offs between competing sustainability factors.
3. Evaluate which sustainability‐related factors consumers report using when making food purchasing decisions.
4. Determine which demographic characteristics and sustainability attitudes are associated with sustainable purchasing behaviors.

## 2 Materials and Methods

### 2.1 Participant Recruitment

Participants were recruited through Prolific, an international online platform through which researchers can advertise paid survey studies to platform users. Interested participants who were over 18 years of age and had USA residency were eligible. The Prolific platform option of recruiting a “representative sample” was selected so that the demographic distribution of our participants was similar to that of USA census data, considering sex, age, and ethnicity. Eligible participants were shown a description of the study before being redirected to RedJade (RedJade Sensory Solutions LLC, Pleasant Hill, CA, USA), where the survey was designed and implemented. This protocol was reviewed and granted exempt status by the Michigan State University Institutional Review Board (STUDY00012099), and participants gave informed consent before completing the survey. Participants who did not complete the survey or provide consent were excluded from analysis, resulting in a final sample of *N* = 475 consumers (Figure 1). Participants were compensated for participating in the study.

### 2.2 Survey Design

A literature review was conducted to analyze existing research on sustainability, food waste, food purchasing habits, current sustainability surveys, and other sustainability topics. Each section of the survey was iteratively developed and tested by members of the research team. The final survey questionnaire consisted of four sections assessing sustainability‐related beliefs, attitudes, and behaviors, and concluded with a demographic questionnaire (Figure 1).

#### 2.2.1 Maximum Difference (MaxDiff) Test

Participants were shown a list of five product characteristics and asked to select which characteristics they deemed to be the most and least important for sustainability from the list. Participants were shown 12 sets of five characteristics randomly chosen from a list of 19 sustainability‐related characteristics. The chosen attributes were grouped into four general categories: product packaging, product certifications or claims, the ingredients or processing used to make the product, and the product size and waste. Product packaging attributes included recyclability, reusability, compostability, minimal packaging, or no packaging. Product certifications or claims included fair trade, certified vegan, non‐GMO, certified humane, USDA organic, reduced carbon footprint, and locally produced products. Ingredient and processing attributes included minimally processed, “clean‐label”, plant‐based, includes preservatives, and processed to extend shelf life. The last category covered products purchased in bulk and products containing the exact amount needed for a use with no wasted food. The full list of attributes is provided in Table S1.

#### 2.2.2 Sustainability Tensions Scenarios

Participants were shown 10 different scenarios of product choices that represent a tension between competing sustainability factors and asked to choose which of the two products they thought was more sustainable. Each scenario consisted of two products where one was more sustainable in one aspect, while the other was more sustainable in a different aspect. For example, one product may have more sustainable packaging while the other has more sustainable contents. Complete scenario descriptions are shown in Table S2.

#### 2.2.3 Consumer Purchasing Decisions

Participants were shown 11 purchasing criteria and asked to rate on a 4‐point Likert scale (1‐ “Never do this”, 2‐“Occasionally do this”, 3‐“Sometimes do this”, and 4 ‐ “Always do this”) how often they make purchasing decisions based on the criteria. Statements asked how often consumers choose products based on price, taste, smaller product sizes to avoid food waste, larger sizes to avoid packaging waste, local products versus national brands, organically grown products over conventionally grown, replacing meat and dairy with plant‐based options, and whether they recycle product packaging or if they reuse packaging. The full list of purchasing decisions is shown in Table S3.

#### 2.2.4 Packaging Sustainability Attitudes

Participants were asked to rate their agreement with 22 sustainability statements, or attitudes, as presented in Lan et al. (2023). The 22 statements were organized by Lan et al. (2023) into six categories:

1. Attitudes toward purchasing behaviors of products with environmentally friendly packaging.
2. Social influence of using environmentally friendly packaging.
3. Consumers’ perception of packaging effectiveness.
4. Personal image concerns when participating in environmental protection.
5. Quality of environmentally friendly packaging.
6. Intention to buy environmentally friendly packaging.

Participants used a 7‐point Likert scale from 1‐ “Strongly disagree” to 7‐ “Strongly agree”. Statements and their categories are included in Table S4.

#### 2.2.5 Demographics

Participants answered demographic questions about their age, gender, race, education level, household income, number of people living in their household, whether they were the primary grocery shopper of their household, and their zip code. Demographic information is included in Table 2.

### 2.3 Calculation of Estimated CO_2_ Emissions

To provide some perspective on actual carbon dioxide emissions of some of the food products included in the sustainability tensions section of the survey, carbon emissions were estimated using My Emissions (https://myemissions.co/, My Emissions Ltd 2025, London, England). Six of the 20 products were selected for calculation; CO_2_ emissions for the remaining 14 products could not be calculated accurately due to limitations of the software. The ingredients, packaging, and their amounts that were inputted into My Emissions for calculation are included in Table 1 below. While the sustainability of a product is complex and multifactorial, estimated CO_2_ emissions will be used as a proxy for product sustainability in this study to allow for direct quantitative comparison between products.

**TABLE 1:** Product information provided to My Emissions for calculation of CO_2_ emissions.

Product	Food	Packaging	Amount of product
Whole head of cabbage with no packaging	Cabbage	None	2 pounds
Half a head of cabbage packaged in plastic	Cabbage	Pallet wrap, plastic	1 pound
A plastic jug of milk. Recycling is NOT an option.	Cow milk, skimmed	Juice bottle with lid, both PET	1 L
A glass bottle of milk. Recycling is NOT an option.	Cow milk, skimmed	Jar, glass and lid metal	1 L
Plant‐based sausage packed in a plastic tray.	Plant based sausage	Tray and lid, both plastic	1 pound
Pork sausage from the deli counter wrapped in paper.	Pork sausage meat	Wrap, wax greaseproof paper	1 pound

Abbreviation: PET, polyethylene terephthalate.

**TABLE 2:** Sociodemographic characteristics and distribution of survey participants.

N = 475	
Variable	**n (%)**
Gender	
Female	237 (49.9%)
Male	229 (48.2%)
Non‐binary	9 (1.9%)
Age	
18–24	54 (11.4%)
25–34	86 (18.1%)
35–44	80 (16.8%)
45–54	73 (15.4%)
55–64	122 (25.7%)
65+	60 (12.6%)
Education	
Some High School	7 (1.5%)
High School Graduate or Equivalent	62 (13.1%)
Some College	89 (18.7%)
Trade, Technical, or Vocational Training	22 (4.6%)
Associate's Degree	43 (9.1%)
Bachelor's Degree	160 (33.7%)
Master's Degree	66 (13.9%)
Professional Degree	14 (2.9%)
Doctorate Degree	12 (2.5%)
Income	
Under $20,000	50 (10.5%)
$20,000 – $39,999	100 (21.1%)
$40,000 – $59,999	80 (16.8%)
$60,000 – $79,999	72 (15.2%)
$80,000 – $99,999	45 (9.5%)
$100,000 and over	128 (26.9%)
Number of people in household	
1	93 (19.6%)
2	142 (29.9%)
3	112 (23.6%)
4	80 (16.8%)
5	35 (7.4%)
6 or more	13 (2.7%)
Primary shopping responsibility in household	
Primary shopper	307 (64.6%)
Not primary shopper	42 (8.8%)
Shared responsibility	126 (26.5%)
Race^a^	
African American or Black	77 (16.2%)
American Indian or Alaskan Native	21 (4.4%)
Asian	36 (7.6%)
Caucasian or White	369 (77.7%)
Hispanic, Latino/a, or Spanish Origin	68 (14.3%)
Native Hawaiian or Pacific Islander	5 (1.1%)

^a^ Participants were able to select multiple race identities.

### 2.4 Data Analysis

Data analysis was conducted using R v4.3.2 with RStudio v2025.05.0 (Posit team 2025). The Tidyverse v2.0.0 (Wickham et al. 2019) collection of packages formed the core framework for data manipulation and visualization. Survey data was imported with readxl v1.4.3 (Wickham and Bryan 2025), with additional data cleaning done with janitor v2.2.1 (Firke 2024). Graphical representation of participant locations was conducted using zipcodeR v0.3.5 (Rozzi 2021) and visualized with maps v3.4.3 (Becker et al. 2025). To support formatting and labeling in some plots, ggstats v0.9.0 (Lamarange 2025) and patchwork v1.3.2.9000 (Pederson, 2025) were used. Standardized beta coefficients were calculated using QuantPsyc v1.6 (Fletcher 2022).

#### 2.4.1 Analysis of Perceived Importance of Sustainability Characteristics

Data from the MaxDiff questionnaire were analyzed by subtracting the percentage of trials for which an attribute was selected as “least important” from the percentage of trials for which it was selected as “most important” to generate an aggregate importance score.

#### 2.4.2 Analysis of Sustainability Tensions

The data from the sustainability tensions section of the survey was analyzed via binomial tests in which the frequency of responses for each of the paired purchase options were evaluated. Purchase options that were selected by a significantly larger number of participants (*α* = 0.05) are denoted in the corresponding figure.

#### 2.4.3 Analysis of Consumer Purchasing Decisions

Participant responses on how often they make purchasing decisions based on a set of criteria were converted to percentages and visualized, with responses grouped by the top two boxes (“always”, “sometimes”) and bottom two boxes (“occasionally”, “never”).

#### 2.4.4 Analysis of Demographics and Sustainability Attitudes on Purchasing Decisions

Participant responses to individual items on the sustainability attitudes questionnaire were summed for each of the six subcategories as outlined in Lan et al. (2023) and Section 2.2.4. to generate participant agreement scores. For sustainability attitudes that were negatively coded, the responses were reversed before analysis. Multiple linear regression (MLR) was conducted to determine the effect of demographic characteristics and sustainability attitudes on purchasing decisions, through generation of beta coefficients. Beta coefficients are presented only for variables with a significant effect on purchasing decisions (*α* = 0.05).

## 3 Results

A total of 475 participants, 49.9% female, completed the questionnaire and met all eligibility criteria. Participants were recruited from across the United States, providing a broad and diverse demographic sample of the U.S. adult population. The average age of participants was 45.7 years (SD = 15.6 years). Income levels were also diverse, with a plurality of participants earning $100,000 or more (26.9%). Most of the survey participants were the primary shoppers of their household (64.6%); additionally, 26.5% shared the responsibility of shopping. Therefore, 91.2% of participants were actively involved in purchasing decisions for their household.

### 3.1 Perceived Importance of Sustainability Characteristics

Consumer responses to the MaxDiff questionnaire revealed that consumers do not value product attributes equally, as they generally view characteristics relating to packaging and locality as more important for sustainability (Figure 2), while the use of preservatives and other certifications are not viewed as very important. The MaxDiff questionnaire showed distinct differences in how consumers view the sustainability of various product characteristics (Figure 2). Local production is the most important aspect for consumers as a sustainability characteristic, suggesting consumers associate sustainability with products made in their area. This was closely followed by recyclable packaging and compostable packaging, highlighting the perceived importance of sustainable disposal of packaging. Interestingly, only two attributes of the food product itself were chosen as important more than not, being made with plant‐based ingredients and minimally processed. Attributes of packaging; reusable, compostable, recyclable, reduced carbon footprint, and local production, were also selected as more important to sustainability than not. All other attributes relating directly to the food inside the packaging were more often selected as least important. Certified vegan products were ranked as the least important for sustainability, followed by using preservatives and processing methods used to extend shelf life. Non‐GMO Project verification and Fair‐Trade certification were also not important to consumers. Aspects that ranked below, but near zero, included USDA organic certification, products with no packaging, smaller sizes of products, and clean label ingredients.

**FIGURE 1:**
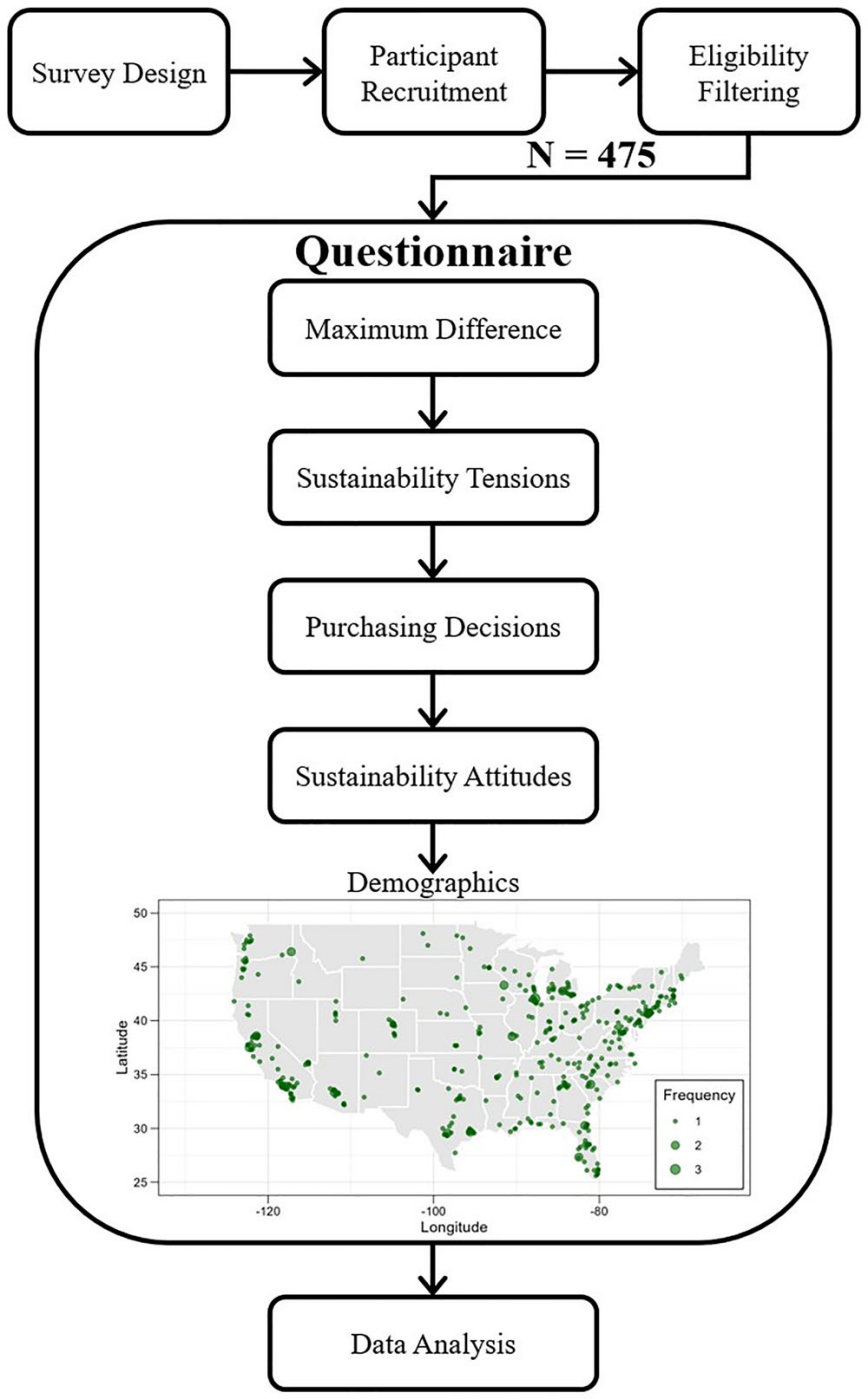
Methodology flowchart. The map shows the distribution of survey participants. Four participants outside the continental U.S. (Hawaii) were excluded from the map for clarity.

**FIGURE 2:**
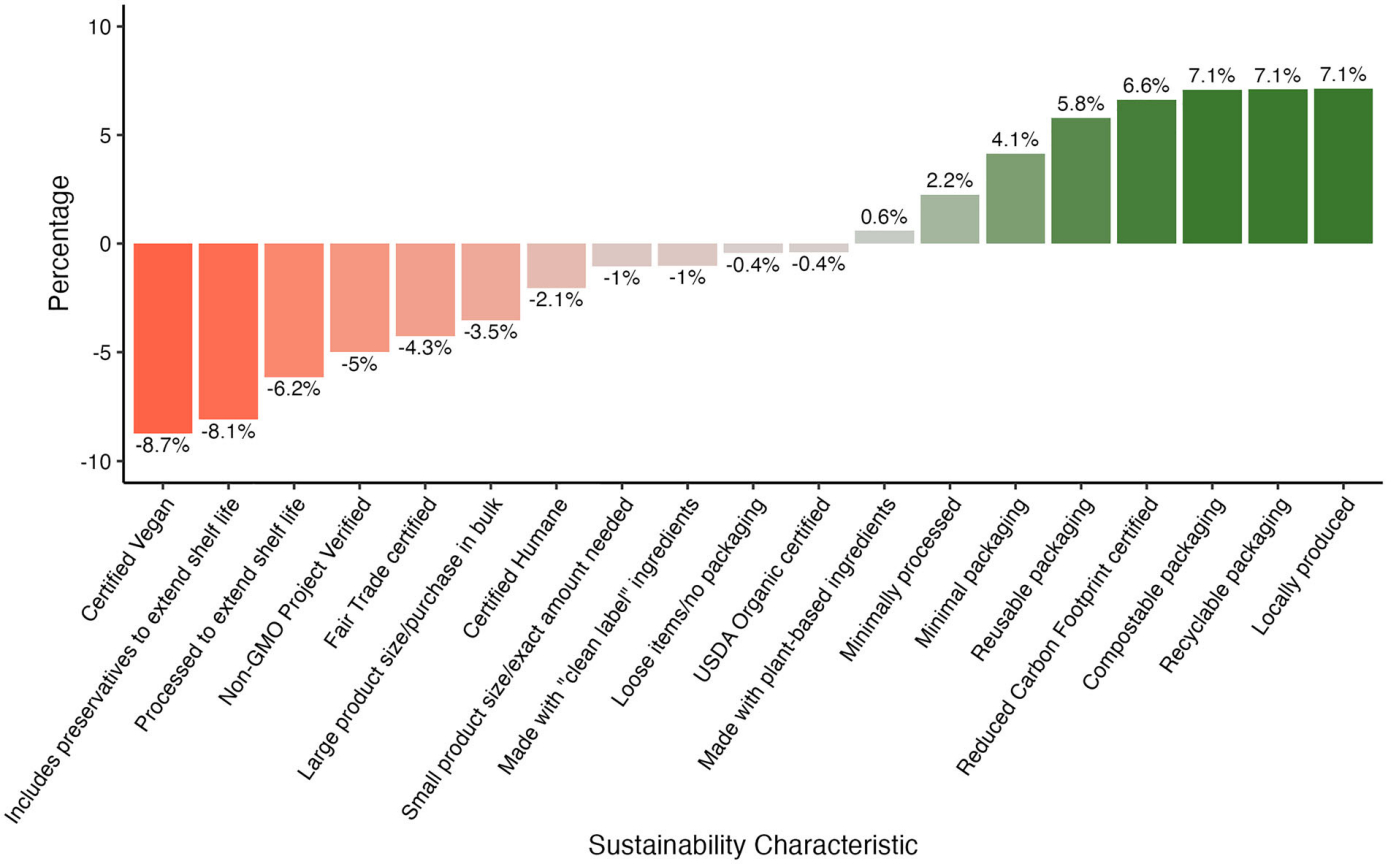
Net difference in percentage of most important and least important selections of sustainability characteristics among all participants.

### 3.2 Sustainability Tensions

Similar to product characteristics, when faced with decisions in the grocery store, consumers focus on packaging and locality. To further understand the perceptions of consumers, a binomial test (*α* = 0.05) was conducted on each scenario to identify scenarios where one option was selected significantly more often as more sustainable than the other. Seven of the 10 scenarios had significance, as indicated in Figure 3A. While packaging is a key factor in consumer sustainability perception, glass is perceived as more sustainable than plastic, and consumers believe they should be avoiding plastic. A glass bottle of milk was seen as more sustainable than a plastic jug of milk (81%) in scenarios where recycling the packaging was an option and when recycling was unavailable. Local strawberries grown with conventional agricultural practices were seen as more sustainable (80%) than organically grown strawberries from a national brand. Additionally, consumers chose yogurt and sausage products in paper packaging, 76% and 71%, respectively, as more sustainable than the same products in plastic packaging.

**FIGURE 3:**
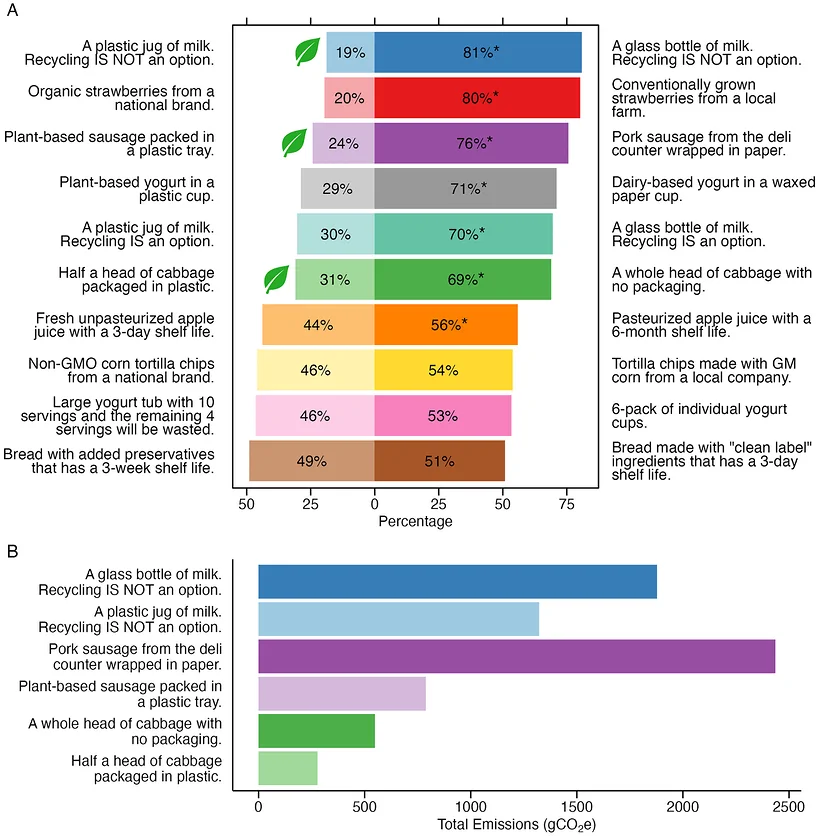
(A) Responses to real‐world sustainability tensions. Dark shading indicates the more often selected choice. Asterisks indicate significantly more often selected, and leaf icons indicate actual lower carbon emissions for selected products. P‐values for these comparisons are included in Table S2. (B) Total estimated emissions of selected products as grams of CO_2_ equivalents. Dark shading indicates the choice more often selected as “more sustainable”.

Consumers also viewed purchasing a product with an excess of servings and no packaging (69%) as more sustainable than a smaller amount with some packaging, but no food waste. This indicates that the environmental harms of packaging waste are more salient to consumers than food waste. Consumers were more evenly divided on the sustainability of products containing genetically modified organisms (GMOs), products with shelf life extending preservatives or processing methods, and purchasing individual serving products with packaging waste or a larger product with food waste.

For the scenarios with calculated CO_2_ emissions, consumers chose the option with higher carbon emissions for all three scenarios selected. Those being whole unpackaged versus a half, packaged head of cabbage; a glass of milk with recycling or no recycling available; and plant‐based sausage versus animal‐based pork sausage with varying packaging types. The cabbage scenario had the least amount of carbon emissions of the set, with both options being less than 600 g CO_2_ equivalents. The pork sausage wrapped in paper had the highest emissions of the set at nearly 2500 g CO_2_ equivalents.

### 3.3 Consumer Purchasing Decisions

When making everyday choices, taste and price are the greatest drivers for consumers to purchase a product, while choosing plant‐based alternatives is the least impactful. Results are shown in Figure 4. A combined 76% of consumers “sometimes” or “always” prioritize taste more often than all other attributes, followed closely by price at 72% of consumers. Recyclable packaging followed closely, with 67% and 63% of consumers opting for a smaller package size to minimize food waste. Additionally, consumers tend to choose locally produced products over national brands, with 60% saying they “sometimes” or “always” choose local. Conversely, replacing meat or dairy products with plant‐based alternatives was the least frequent choice, with 44% and 46% of consumers saying they “never” swap for plant‐based alternatives.

**FIGURE 4:**
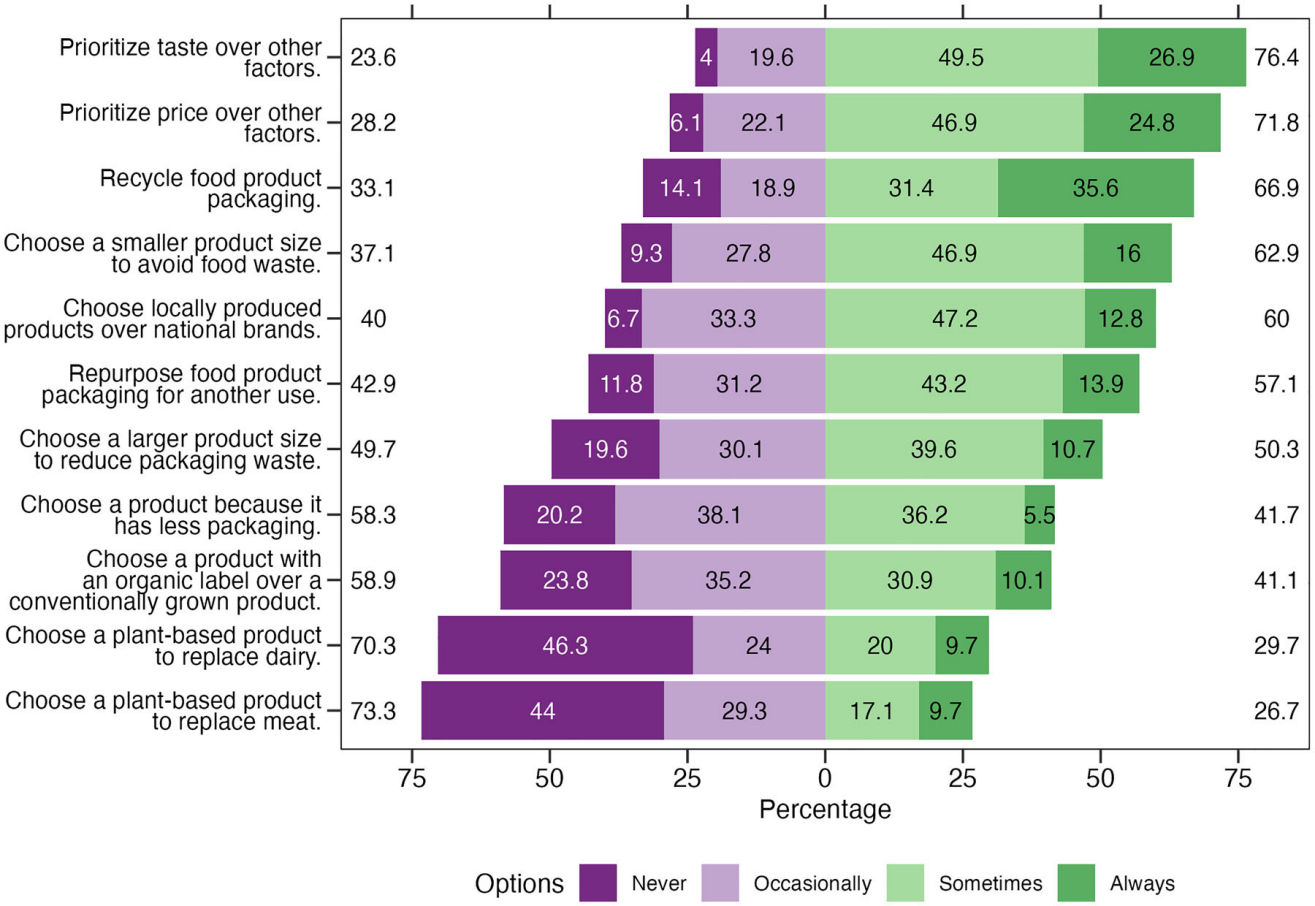
Frequency in responses to purchasing decisions. Percentage of “Never” and “Occasionally” responses were summed and included on the left edge of the plot and responses of “Sometimes” and “Always” were summed and shown on the right edge of the plot. The percentage of each response is shown inside the correspondingly colored bar, with the purchasing decision shown to the left of the plot.

### 3.4 Effects of Demographics and Sustainability Attitudes on Purchasing Decisions

To better understand what factors influence consumer purchasing decisions, beta coefficients were computed using multiple linear regression (MLR) with a confidence interval of 95% (*α* = 0.05). Values are shown in Table 3. Neither gender nor age showed significant influence toward consumer's purchasing decisions and were not included in Table 3. The strongest predictor of purchasing decisions was the intention to buy environmentally friendly packaging, followed by the quality of environmentally friendly packaging, income level, and the consumer's belief in their personal effectiveness in environmental protection. Despite this, concerns of personal image relating to environmental protection participation were the only attitude factor that showed no significance in predicting purchasing decisions.

**TABLE 3:** Purchasing decisions predicted by sustainability attitudes, education, income, household size, and if the participant is their household's primary shopper. Multiple linear regression (*n* = 475). Values in bold are different from zero at a significance level of *p* < 0.05. Green and red shading signify positive and negative correlations, respectively.

	Age	Gender	Education	Income	Household size	Primary shopper	Attitude toward packaging	Social influence	Consumer effectiveness perception	Personal image concern	Packaging quality	Intention to buy	Adjusted *R* ^2^	Model significance
**Choose less packaging**	−0.005	0.011	0.011	0.043	−0.010	−0.054	**−0.208**	0.107	0.067	−0.026	0.042	**0.495**	0.227	<0.001
**Recycle packaging**	0.004	0.034	0.047	**0.108**	−0.022	−0.078	0.004	0.124	0.015	−0.004	**0.136**	**0.184**	0.17	<0.001
**Repurpose packaging**	0.046	−0.009	**0.148**	**−0.118**	**0.131**	**−0.117**	−0.125	0.128	**0.173**	0.008	**0.176**	0.057	0.169	<0.001
**Choose plant‐based over meat**	0.001	−0.025	**0.145**	**0.102**	0.02	**−0.107**	−0.147	0.079	0.069	0.09	**0.155**	0.147	0.168	<0.001
**Choose plant‐based over dairy**	0.004	−0.046	0.085	0.096	−0.036	−0.083	−0.120	0.08	0.011	0.084	**0.14**	0.147	0.107	<0.001
**Choose smaller size to avoid food waste**	0.011	−0.061	0.027	**0.123**	−0.019	0.085	0.024	0.038	0.033	0.008	0.017	**0.18**	0.088	<0.001
**Choose larger size to avoid packaging waste**	0.076	0.028	0.028	0.076	0.013	−0.073	−0.074	**0.255**	0.016	−0.027	−0.026	**0.218**	0.135	<0.001
**Choose organic over conventional**	0.034	−0.057	0.069	0.092	−0.016	−0.058	−0.078	0.128	**0.185**	0.051	−0.031	0.144	0.157	<0.001
**Choose local over national**	0.071	−0.004	0.064	0.056	0.019	−0.049	**−0.178**	**0.136**	**0.163**	0.032	−0.001	**0.261**	0.166	<0.001
**Prioritize price over other factors**	−0.075	−0.040	0.086	−0.077	−0.008	−0.001	0.129	−0.088	**−0.203**	−0.013	0.044	−0.005	0.023	0.029
**Prioritize taste over other factors**	0.046	−0.054	0.092	0.063	−0.036	−0.062	0.122	0.009	0.006	−0.023	0.108	−0.174	0.019	0.045

Consumer sustainability behaviors are linked with some demographic characteristics. Higher‐income consumers tend to recycle food packaging more often and choose smaller sizes of products to reduce food waste but are less likely to repurpose food packaging. In addition, consumers with higher income and higher education were more likely to choose a plant‐based alternative instead of meat. Moreover, primary shoppers are less likely to choose plant‐based over meat.

Participants with larger households or higher education are more likely to repurpose food packaging, while those who are primary shoppers are less likely to repurpose food packaging. Interestingly, those who feel that using products with environmentally friendly packaging will positively affect the environment are actually less likely to choose products with less packaging or locally produced products. Alternatively, those who feel pressure from their social circles to use environmentally friendly packaging are more likely to choose local products and products with larger sizes to reduce packaging waste.

As consumers’ perception of environmentally friendly packaging effectiveness increases, they are more likely to repurpose packaging and choose organic and local products over conventionally grown produce from national brands. Those who view environmentally friendly packaging to be of equal quality as conventional packaging recycle packaging, repurpose packaging, and choose plant‐based alternatives to meat and dairy products more often. As consumers’ intention to buy products with environmentally friendly packaging increases, they choose products with less packaging and recycle packaging more often. They are also more likely to choose larger sizes of products to reduce packaging waste while also choosing smaller sizes of products to reduce food waste more often. These same consumers also choose local products more often.

## 4 Discussion

This study was designed to determine the extent of consumers’ attention to and knowledge of sustainable food products and packaging and whether the sustainability attributes of a product influence their stated purchasing decisions. While consumers express a clear desire to make sustainable choices, their decision‐making prioritizes easily understood factors, like packaging materials, instead of factors that are potentially more impactful, like food waste and protein source.

Across all survey sections, consumers perceived glass and paper packaging as more environmentally sustainable than plastic packaging, as similarly reported in Dlamini et al. (2024) and Thomas et al. (2025). This preference for glass and the belief in its superior sustainability holds true, even when the option to recycle is unavailable. This may be related to the view that glass packaging is more reusable than other packaging types. However, while Miao et al. (2023) found that most consumers exhibited a high willingness to adopt reusable packaging practices, they were generally held back by the inconvenience of reusing containers. Additionally, the success of reusable packaging hinges on preexisting habits, wherein recycling is a much more common habit among consumers than reusing. Furthermore, 85% of consumers want to purchase products with reusable packaging, but only 16% are reusing product packaging (Poole 2019). It is commonly known that glass is infinitely recyclable, yet it is heavier and therefore more energy‐intensive to transport than paper or plastic alternatives. According to the US EPA (2020), only 31.3% of glass packaging produced in the United States was recycled compared to 80.9% of paper and paperboard packaging and 13.6% of plastic packaging. The popularity of single‐use plastics likely decreases consumer perception of the sustainability of plastic packaging, which can also be attributed to the low recycling rate compared to glass and paper. The stigmatization of plastic, commonly referred to as “plastic bashing,” is the result of highly publicized negative impacts of plastic waste in the environment, like polluted ocean beaches and microplastic residues. This stigmatization may further demonize plastic packaging, despite its benefits in weight and emissions.

Consumers also tend to view products as more sustainable when their packaging is recyclable, reusable, or compostable, regardless of product characteristics that may dwarf sustainability differences in packaging. For the three sustainability tensions evaluated for carbon emissions, survey participants overwhelmingly chose the least sustainable option for all three scenarios. When presented with an unpackaged product with excessive servings, leading to potential food waste, and a smaller, packaged product, preventing food waste, 69% of consumers chose the unpackaged option. Our study suggests that plastic waste is of significantly greater concern than food waste to consumers, even though food waste is responsible for 58% of methane emissions from landfills (Krause et al. 2023).

While participants ranked shelf‐life extension, through both ingredient use and processing methods, as among the least important aspects of sustainability, spoilage remains a primary driver of food waste. Food waste itself carries substantial environmental consequences that are often underestimated by consumers. In the United States, 31% of the available food supply at the retail and consumer level went to waste, with consumer losses accounting for 21% of the total, resulting in 133 billion pounds of wasted food worth $161.6 billion (Buzby et al. 2014). This pattern is not one of ignorance, as consumers often recognize that food waste is a problem, but tend to focus the blame on external factors and not their own household contributions (Brennan et al. 2021).

This discrepancy is consequential because it limits consumer motivation to seek out and value preservation solutions. By extending the windows of quality and safety with preservatives, this technology can help reduce food waste associated with the inefficiency of supply chains and the unpredictability of household consumption. As Martindale (2014) demonstrated in a British study, households wasted 47% more fresh food than frozen, and the carbon footprint of frozen food per household was just 70% that of typical fresh food consumption. When consumers dismiss preservation to reduce food waste, they may be choosing products with higher environmental costs. This perception gap is compounded by the fact that public sustainability campaigns have historically focused on recycling, rather than food waste reduction (Barnett et al. 2023), leaving consumers without understanding the full scope of where their food waste fits into the broader sustainability picture. Closing this gap requires education efforts that focus on the environmental costs of food waste and reframing preservation methods as a tool for sustainability.

Despite the scientific consensus on the environmental benefits of plant‐based diets, our results reveal significant consumer resistance to plant‐based alternatives. “Certified Vegan” was ranked as the least important sustainability characteristic, and plant‐based ingredients were rated as barely net positive. This resistance may stem from a complex of sensory, cultural, and economic barriers that together outweigh sustainability factors. Consumers’ preferences for packaging materials add another competing factor, as our sustainability tensions data illustrates vividly. 76% of participants chose pork sausage wrapped in paper as more sustainable than plant‐based sausage in a plastic tray, effectively prioritizing packaging material over protein source despite the much higher carbon footprint of the pork product. Similar findings were reported by Otto et al. (2021), who reviewed European perceptions of food packaging and found that consumers systematically attribute sustainability to the packaging material, rather than the food inside. Additionally, Giacone et al. (2024) found that consumers underestimated the negative environmental impact of dairy products and considered them to be more environmentally friendly than plant‐based alternatives.

In our study, plant‐based options were pitted against packaging considerations, but resistance to plant‐based products is well‐documented even without such tensions. Cultural attachment to meat, sensory expectations, and concerns about processing and nutritional quality are all established barriers to widespread acceptance (Rockström et al. 2025). The Planetary Health Diet (PHD) proposed by Rockström et al. (2025) offers a diet that supports optimal nutrition and sustainability goals. Rather than complete elimination of animal products, the PHD emphasizes gradual shifts toward plant‐forward eating that may be more achievable for the large portion of consumers who report never choosing plant‐based options (44%–46% in our sample). Messaging that acknowledges consumer preferences while highlighting environmental benefits of plant‐forward eating may be more effective than sustainability framing alone.

Understanding who makes sustainable choices and why is just as important as identifying what those choices should be, and our regression analysis offers meaningful guidance for designing targeted sustainability interventions. The strongest predictors of sustainable purchasing behavior were not demographic characteristics like age or gender, but attitudinal ones. Intention to buy environmentally friendly packaging and perceived consumer effectiveness of environmentally friendly packaging were among the most consistent drivers. This aligns with broader literature showing that consumers’ belief in the effectiveness of their actions toward sustainability is a stronger predictor of sustainable behavior than awareness alone (Vermeir and Verbeke 2006).

Packaging‐specific attitudes were also especially predictive in our data. Consumers who believe that environmentally friendly packaging is of equal quality to conventional packaging were more likely to recycle and repurpose, suggesting that quality perception is a key driver of action. This is consistent with findings from Hao et al. (2019), who found that consumers’ willingness to pay for green packaging was significantly shaped by their perceptions of its quality. Similarly, Ketelsen et al. (2020) concluded that closing the gap between awareness and actual purchasing behavior requires targeting communication that addresses both the environmental impact and quality of sustainable packaging options.

The role of social influence in purchasing behavior is also noteworthy. Consumers who felt social pressure from their peers to use environmentally friendly packaging were significantly more likely to choose locally produced products, suggesting that sustainable purchasing can operate as a social signal reinforced by community norms. Research on packaging‐free retail systems has shown similar dynamics. Jiménez Romanillos et al. (2024) found that consumer behavior in packaging‐free systems was strongly shaped by social norms, convenience, and normalization of those practices in consumers’ social environments.

Finally, convenience remains an underappreciated barrier. Higher‐income consumers in our sample were more likely to recycle, but less likely to reuse packaging, reflecting that recycling is a routine embedded in household infrastructure while reuse demands deliberate effort. Coelho et al. (2020) reached a similar conclusion in their review of sustainable packaging systems, finding that even when consumers expressed preference for reusable options, adoption of reusable packaging depended on the convenience and availability of collection, return, and refilling systems. Furthermore, it is important for companies to understand that packaging is a major sustainability cue for consumers and to capture positive consumer preferences for packaging that aligns with other sustainability traits of the product. Together, these findings suggest that sustainability education may benefit from addressing the attitudes and perceptions associated with sustainable purchasing behaviors, particularly perceptions of packaging effectiveness, quality, and personal environmental impact. These findings also highlight the potential value of communicating sustainability beyond packaging to help consumers consider other attributes of food products and their environmental impacts.

## 5 Conclusion

To meet global sustainability goals, sustainability education must broaden its narrow focus on recycling to the total lifespan of a product and its packaging. This study revealed significant gaps in consumer perceptions of sustainability and the true impacts of food products and packaging. While consumers consistently prioritize packaging‐related attributes like recyclability and material type, these are not always the most impactful factors in terms of carbon emissions. Our findings demonstrate that consumers frequently misidentify more sustainable options, particularly in situations involving food waste, preservation methods, and plant‐based alternatives. Consumers’ over‐emphasis on packaging and underestimation of factors like food waste and protein source suggests that current sustainability messaging can be incomplete, confusing, and at times misleading. In addition to promoting recycling, increasing targeted consumer education on less familiar areas of sustainability like protein sources, packaging types, and preservation methods is needed to shift consumer behavior and dramatically improve the sustainability of our food system.

## Author Contributions

**Jonas Ahonen**: investigation, writing – original draft, visualization, formal analysis, methodology. **Anna Wagner**: methodology, writing – review and editing. **Hannah Mulheron**: methodology, project administration, writing – review and editing. **Alissa Nolden**: conceptualization, methodology; writing – review and editing. **Emily J. Mayhew**: conceptualization; writing – review and editing; methodology; supervision.

## Conflicts of Interest

The authors declare no conflicts of interest.

## Supporting information




**Supplementary Material**: jfds71511‐sup‐0001‐SuppMat.DOC

## Data Availability

Anonymized data and analysis scripts will be made available upon reasonable request.
