# Supplementary material for: What Makes a Product Sustainable? A US‐Based Consumer Survey Finds That Consumers Overemphasize the Impact of Packaging on Product Sustainability
Source: J Food Sci. 2026 Oct 10;91(10):e71511. doi: 10.1111/1750-3841.71511 (PMC13654058; doi:10.1111/1750-3841.71511)
Supplement: Supplementary file 1 [file JFDS-91-0-s001.DOC]

**Supplementary Text**

**Table S1.** Maximum Difference Test Attributes

| Category | Attribute |
| --- | --- |
| Packaging | Recyclable packaging |
| Packaging | Reusable packaging |
| Packaging | Minimal packaging |
| Packaging | Loose items/no packaging |
| Packaging | Compostable packaging |
| Product certifications or claims | Fair Trade certified |
| Product certifications or claims | Certified Vegan |
| Product certifications or claims | Non-GMO Project verified |
| Product certifications or claims | Certified Humane |
| Product certifications or claims | USDA Organic certified |
| Product certifications or claims | Reduced Carbon Footprint certified |
| Product certifications or claims | Locally produced |
| Amount of processing | Minimally processed |
| Amount of processing | Made with “clean label” ingredients |
| Amount of processing | Made with plant-based ingredients |
| Amount of processing | Includes preservatives to extend shelf life |
| Amount of processing | Processed to extend shelf life |
| Product size | Small product size/exact amount needed |
| Product size | Large product size/purchased in bulk |

**Table S2.** Sustainability tensions scenario prompts.

| Attribute to Compare | Scenario | Choice 1 | Choice 2 | p-value |
| --- | --- | --- | --- | --- |
| Food Waste vs.  Packaging Waste | You need exactly half a head of cabbage for a recipe and have no use for leftovers. | Half a head of cabbage packaged in plastic. | A whole head of cabbage with no packaging. | <0.001 |
|  | You need 6 servings of yogurt for a breakfast event. | 6-pack of individual yogurt cups. | Large yogurt tub with 10 servings and the remaining servings wasted. | 0.142 |
| Plastic vs. Glass | You need a gallon of milk. Recycling the packaging IS NOT an option. | A glass bottle of milk. | A plastic jug of milk. | <0.001 |
|  | You need a gallon of milk. Recycling the packaging IS an option. | A glass bottle of milk. | A plastic jug of milk. | <0.001 |
| Plant-based Product vs. Packaging Waste | You need a pound of sausage for a recipe. | Plant-based sausage packed in a plastic tray. | Pork sausage from the deli counter wrapped in paper. | <0.001 |
|  | You are choosing a yogurt product to purchase. | Plant-based yogurt in a plastic cup. | Dairy-based yogurt in a waxed paper cup. | <0.001 |
| Organic vs. Local | You need a pint of strawberries. | Organic strawberries from a national brand. | Conventionally grown strawberries from a local farm. | <0.001 |
|  | You need a bag of corn tortilla chips for a party. | Non-GMO corn tortilla chips from a national brand. | Tortilla chips made with GM corn from a local company. | 0.098 |
| “Clean label” vs Added Preservatives/Processing | You need a loaf of bread. | “Clean label” bread that has a 3-day shelf life. | Bread with added preservatives that has a 3-week shelf life. | 0.713 |
|  | You need apple juice. | Fresh unpasteurized apple juice with a 3-day shelf life. | Pasteurized apple juice with a 6-month shelf life. | 0.010 |

**Table S3.** Purchasing decisions prompts.

Purchasing Decision

Choose a product because it has less packaging.

Recycle food packaging.

Repurpose food packaging for another use.

Choose a plant-based product to replace meat.

Choose a plant-based product to replace dairy.

Choose a smaller product size to avoid food waste.

Choose a larger product size to reduce packaging waste.

Choose a product with an organic label over a conventionally grown product.

Choose locally produced products over national brands.

Prioritize price over other factors.

Prioritize taste over other factors.

**Table S4.** Sustainability attitudes prompts

| Variable | Description |
| --- | --- |
| Attitudes toward purchase behaviors of products with environmentally friendly packaging | |
| AT1 | Using products with environmentally friendly packaging will reduce environmental pollution and improve the environment. |
| AT2 | Using products with environmentally friendly packaging will help reduce the waste of natural resources. |
| AT3 | Using products with environmentally friendly packaging will help conserve natural resources. |
| AT4 | I feel comfortable using products with environmentally friendly packaging. |
| Social influence | |
| SI1 | My friends think I should use products with environmentally friendly packaging. |
| SI2 | People important to me encourage me to use products with environmentally friendly packaging. |
| SI3 | Information in the media encourages me to try products with environmentally friendly packaging. |
| SI4 | I learned that consuming environmentally friendly packaging products contributes to a better environment. |
| Consumers’ perception of effectiveness | |
| CP1 | I can protect the environment by buying products with environmentally friendly packaging. |
| CP2 | I think that if I do some environmental protection in my daily life, I will contribute a lot to our environment. |
| CP3 | I think if I participate in environmental protection, I will encourage my family and friends to participate too. |
| CP4 | I don't think I can help solve environmental problems. |
| Personal image concerns | |
| PC1 | Participating in environmental protection makes me unique among others. |
| PC2 | I am considered obsolete (outdated) if I do not participate in environmental protection. |
| Quality of environmentally friendly packaging | |
| QL1 | I believe environmentally friendly packaging can protect the product inside. |
| QL2 | I believe that environmentally friendly packaging can be as durable as conventional packaging. |
| QL3 | I think that environmentally friendly packaging can be as beautiful and convey product content as much as conventional packaging. |
| QL4 | I think environmentally friendly packaging can attract consumers' attention as much as conventional packaging. |
| Intention to buy products with environmentally friendly packaging | |
| IB1 | I want to buy products with environmentally friendly packaging. |
| IB2 | I want to consume products with environmentally friendly packaging. |
| IB3 | I consider purchasing products with environmentally friendly packaging. |
| IB3 | I want to encourage people to buy products with environmentally friendly packaging. |

**Survey Questionnaire**
